# A computer-driven ventilator liberation protocol in pediatric patients: a single-center pilot randomized controlled trial

**DOI:** 10.3389/fped.2025.1594160

**Published:** 2025-07-18

**Authors:** Song Chen, Changxue Xiao, Xue Lu, Min Liao, Chengjun Liu, Feng Xu, Jing Li

**Affiliations:** Department of Critical Care Medicine, Children’s Hospital of Chongqing Medical University, National Clinical Research Center for Child Health and Disorders, Ministry of Education Key Laboratory of Child Development and Disorders, Chongqing Key Laboratory of Pediatric Metabolism and Inflammatory Diseases, Chongqing, China

**Keywords:** critical care, ventilation, pediatrics, liberation protocol, pilot study

## Abstract

**Objective:**

Timely liberation from invasive mechanical ventilation (IMV) is important. We aimed to determine the feasibility of our study protocol for the conduction of a larger prospective trial to examine the utility of a computer-driven liberation protocol in pediatric patients.

**Design:**

Single-center pilot randomized controlled trial.

**Setting:**

Single, tertiary care, 52-bed, academic pediatric intensive care unit (PICU).

**Patients:**

Patients aged from 28 days to 18 years undergoing IMV for more than 24 h.

**Interventions:**

Patients were randomly assigned to test and control groups in a ratio of 1:1. The test group underwent ventilator liberation driven by a computerized algorithm combining protocolized screening, air leak testing, and spontaneous breathing testing. The control group underwent ventilator liberation driven by the attending physician according to standard care.

**Measurements and main results:**

A total of 40 patients (20 in each group) were randomized. Baseline characteristics of the two groups were similar. Durations of IMV were 95.3 h (95%CI, 9.07–181.53) in the test group and 113.3 h (95%CI, 85.90–140.70) in the control group and were similar (*p* = 0.62). PICU length of stay [6.9 days [95%CI, 5.00–8.86] vs. 7.0 days [95%CI, 5.58–8.40]; *p* = 0.74] and hospital length of stay [22.9 days [95%CI, 11.48–34.24] vs. 26.9 days [95%CI, 17.86–35.94]; *p* = 0.31] were similar between the test and control groups, respectively.

**Conclusions:**

Our pilot study suggests that the conduction of a larger prospective trial of a computer-driven ventilator liberation protocol is feasible in our PICU. And a larger trial is needed to further explore the utility of a computer-driven ventilator liberation protocol.

**Clinical Trial Registration:**

https://www.chictr.org.cn/showproj.html?proj=168024, Chinese Clinical Trial Registry ChiCTR2200060033.

## 1 Introduction

Invasive mechanical ventilation (IMV) is an important life support measure in critical care units (1–3), but can be a double-edged sword. Prolonged IMV is significantly correlated with ventilator-associated pneumonia, ventilator-associated lung injury, and airway injury; and is an independent risk factor of poor clinical outcomes (1–7). Thus, timely liberation from IMV after resolution of the primary indication for ventilation is important. However, the identification of a feasible timepoint for IMV liberation is difficult. Up to 50% of patients experiencing unplanned extubation do not require re-intubation, which suggests that physician-driven standard care can often miss the optimal timepoint of liberation (7).

A review of 17 trials comparing protocolized to non-protocolized liberation from IMV disclosed that most studies were centered on spontaneous breathing testing (SBT), and that the use of protocols significantly reduced the duration of ventilation, medical costs, and iatrogenic complications (8). The use of IMV liberation protocols has been recommended in guidelines for adult patients (9, 10). A recent international clinical practice guideline for pediatric IMV liberation has also recommended the use of a protocolized approach (11). Unfortunately, the certainty of evidence was low in the guideline. Although observational and interventional studies exist, most of the pediatric literature consists of narrative reviews and meta-analyses, underscoring the need for high-quality research in this field (11).

Most studies reported that protocolized liberation shortened IMV duration and reduced extubation failure rates (12, 13). Potential drawbacks, however, include increased staff burden and screening fatigue, which may contribute to low protocol compliance (14). Computer-driven liberation protocols can screen vital signs, ventilator parameters, and laboratory data automatically; provide a reliable measure to inform IMV liberation; and can overcome screening fatigue that may lower compliance rates and reduce IMV duration ultimately. The objectives of this pilot randomized controlled clinical trial were to determine the feasibility of applying our computer-driven decision support tool to pediatric patients; the feasibility of conducting a larger prospective trial of our study protocol to explore the efficacy of the intervention; and to train pediatric intensive care unit (PICU) personnel.

## 2 Materials and methods

### 2.1 Study design

We conducted a single-center pilot randomized controlled pilot study in the 52-bed PICU of the Children's Hospital of Chongqing Medical University, China.

The study protocol was approved by the Institutional Review Board of Children's Hospital of Chongqing Medical University (file no. 2022-75; approval date: April 2, 2022). The study was conducted according to CONSORT guidelines, was performed in accordance with the 1964 Declaration of Helsinki and its later amendments or comparable ethical standards, and was registered in Chinese Clinical Trial Registry (ChiCTR2200060033). Written informed consent for participation was obtained from children's parents or legal guardians.

### 2.2 Study participants

Patients aged from 28 days to 18 years receiving IMV for more than 24 h were included. Exclusion criteria were: intubation indicated for upper airway obstruction; or the presence of either airway malformation, diaphragmatic hernia or paralysis, cyanotic congenital heart disease, primary pulmonary hypertension, neuromuscular disease, central respiratory failure, chronic lung disease, tracheostomy, or an imminently fatal prognosis. Chronic lung disease was defined as a condition requiring chronic oxygen therapy before hospitalization, such as bronchopulmonary dysplasia. Patients were enrolled only after the first intubation during their hospitalization. Patients were excluded from the final analysis if they had experienced either IMV for more than 21 days; tracheostomy; interhospital transfer; withdrawal of treatment; in-hospital mortality; or if parents or guardians opted out of the study.

### 2.3 Randomization and allocation masking

Patients were randomly assigned to undergo IMV liberation according to either a computer-driven liberation protocol or usual care in a ratio of 1:1. The allocation sequence, presented in prenumbered and sealed envelopes, was randomly designated as either “test” or “control” by a computer-generated random number table. The computer-generated allocation sequence was completed by a third-party researcher who was not involved in this study. Once a patient entered the study, investigators immediately opened the envelope in order.

### 2.4 Intervention masking

The two study groups were treated by the same medical team. However, medical team members were blinded to patient group assignment until the computer-driven IMV liberation protocol group patients completed protocolized screening.

### 2.5 Intervention

**Figure 1:**
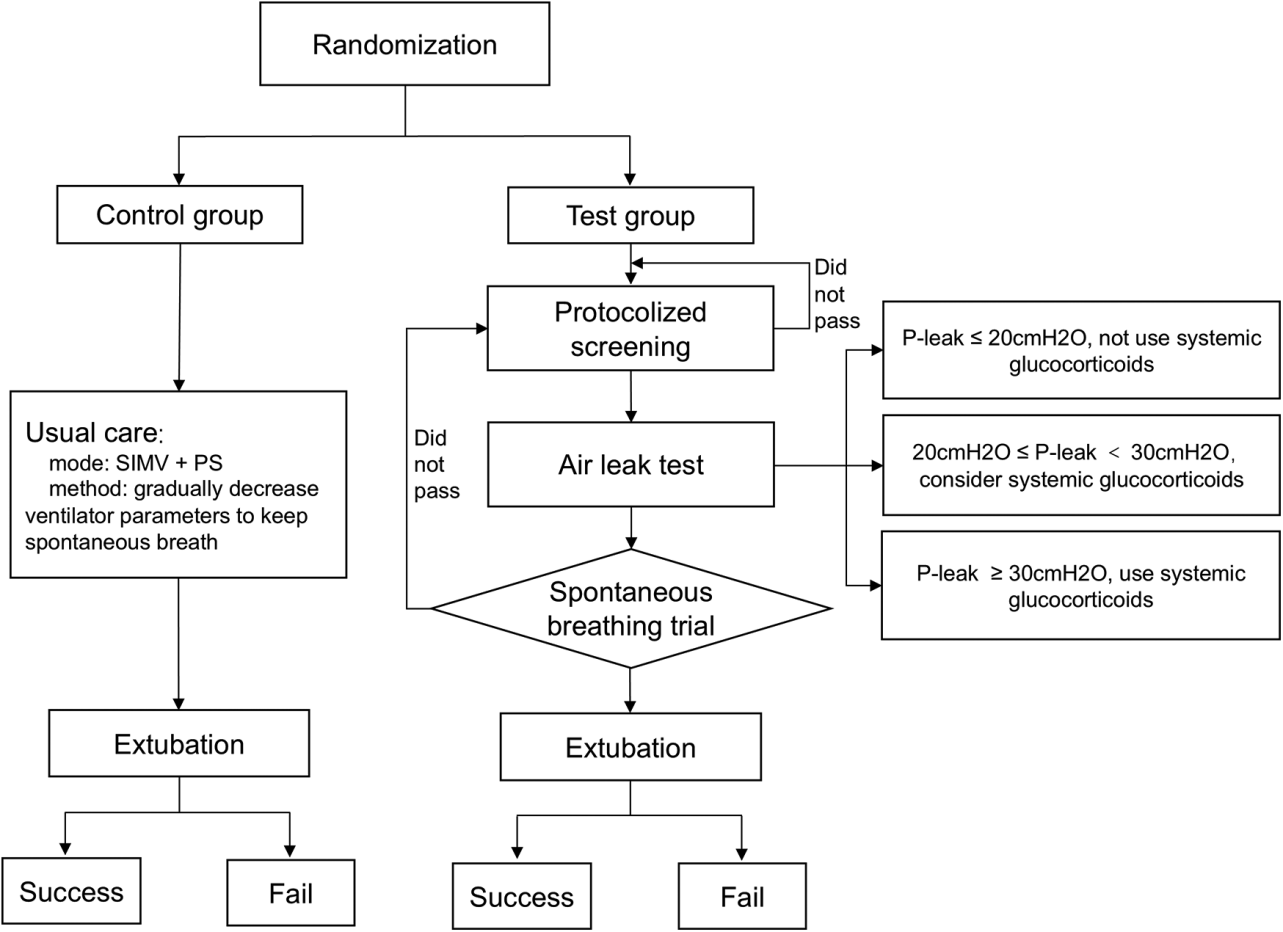
Study design. SIMV, synchronized intermittent mandatory ventilation; PS, pressure support; P-leak, the pressure causing endotracheal peritubal leak.

Our study design is illustrated in Figure 1. Usual (control) care was driven by the attending physician. Synchronized intermittent mandatory ventilation with pressure support was used during standard care. When the patient was recovering from the disease for which intubation was indicated, ventilator parameters were decreased gradually to enable spontaneous breathing.

The test group was driven by a computerized algorithm. The IMV liberation protocol, which combined protocolized screening, air leak testing, and SBT was based on the IntelliSpace Critical Care and Anesthesia decision support tool (ICCA, Koninklijke Philips N.V., The Netherlands). ICCA captured vital signs, ventilator parameters, laboratory data, and treatment measures. Based on those data, ICCA screened patients fit for SBT automatically, and drove the liberation protocol. If a patient had passed the protocol, ICCA would advise the attending physician, who would make final patient management decisions.

ICCA screened all patients at 8:00 AM daily. Investigators were alerted to patients who had met the following criteria: fraction of inspired oxygen (FiO_2_) ≤50%; positive end-expiratory pressure (PEEP) ≤8 cmH_2_O; peak inspiratory pressure (PIP) ≤25 cmH_2_O; state behavioral scale (SBS) = −1 or 0 (15); no continuous use of neuromuscular blockers in the preceding 24 h; stable hemodynamic status (vasoactive-inotropic score [VIS] ≤20; VIS = epinephrine (μg/kg/min) × 100 + norepinephrine (μg/kg/min) × 100 + milrinone (μg/kg/min) × 10 + vasopressin (U/kg/min) × 10,000 + dopamine (μg/kg/min) + dobutamine (μg/kg/min)) (16, 17); and presence of respiratory drive. All the above data were recorded automatically in ICCA or manually by the nursing staff every 2 h.

Screened patients underwent immediate air leak testing. The air leak test, as a predictor of postextubation stridor, was used to guide systemic glucocorticoid therapy (9, 11, 18, 19). Its application has been described in detail (20–22). The air leak test was performed with the patient supine and head in the midline position. After complete deflation of the endotracheal tube cuff, the patient was manually ventilated to a maximal pressure of 30 cmH_2_O. The pressure at which an audible air leak heard was recorded. If air leak pressure was ≤20 cmH_2_O, systemic glucocorticoid therapy was not to be considered; if air leak pressure was >20 cmH_2_O and ≤30 cmH_2_O, systemic glucocorticoid therapy could be considered in the presence of other factors that might cause postextubation stridor; if air leak pressure was ≥30 cmH_2_O, systemic glucocorticoid therapy given 4–6 h prior to extubation was considered (9, 11, 18).

The air leak test was followed by SBT, which simulates postextubation conditions (23, 24). Patients were placed on FiO_2_ of 40%, PEEP of 5cmH_2_O, and pressure support of 5–8 cmH_2_O. The SBT was conducted for 0.5–2 h. The test was interrupted if patients exhibited either SpO_2 <_ 90%; a heart rate increases of more than 20% compared to baseline; signs of tachypnea, paradoxical respiration, or increased respiratory work; or tidal volume ≤5 ml/kg (weight ≥60 kg, tidal volume ≤300 ml) (12, 25–27). An arterial blood gas analysis was performed after 30 min of SBT. If the patient passed the SBT and exhibited body temperature <38.5°C, pH > 7.3, lactate <2 mmol/L, and PaCO_2_ < 55 mmHg, ICCA would advise IMV liberation. The attending physician made final management decision according to patient's clinical status. If the attending physician declined the ICCA recommendation, previous MV parameters would be rebuilt, and the entire process would be repeated the next day.

Both groups received the same sedation protocol. SBS was adopted to assess sedation level. Analgesia with sufentanil (0.04–0.12 ug/kg/h) and sedation with midazolam (1–5 ug/kg/min) were routinely administered to all intubated patients.

### 2.6 Outcomes

Extubation was defined as successful if re-intubation was not indicated within 48 h. The primary outcome was the duration of IMV from initiation until the first extubation. Secondary outcomes were successful extubation frequency, noninvasive respiratory support usage after extubation, PICU length of stay (PICU LOS), and hospital LOS.

### 2.7 Power and sample size calculations

A comparison of a ventilator liberation protocol that combined daily evaluation and SBT to standard care disclosed a median IMV duration of 3.5 days [95% confidence interval (CI), 3.0–4.0] in the test group and 4.7 days (95%CI, 4.1–5. 3) in the control group (*p* = 0.0127) (12). The aforementioned study suggests that a sample size of 375 patients (187 patients for the control group, 188 patients for the test group) would confer adequate statistical power (1-*β* = 80%, two-side *α* = 0.05, accrual time = 20 months, and proportion lost = 5% for each group). Our pilot study included 40 patients (20 in each group), or 10% of the sample size calculated above.

### 2.8 Data analysis

Statistical analysis was performed with SPSS version 26 (IBM Corp, Armonk, NY, USA). Continuous variables were presented as median (IQR). Intergroup differences were evaluated with the Mann–Whitney *U* test. Categorical variables were presented as counts (percentages). Kaplan–Meier survival curves were used to analyze the probability of remaining on IMV, with comparisons based on the log-rank test. A *p*-value < 0.05 was considered statistically significant.

## 3 Results

### 3.1 Patient characteristics

**Figure 2:**
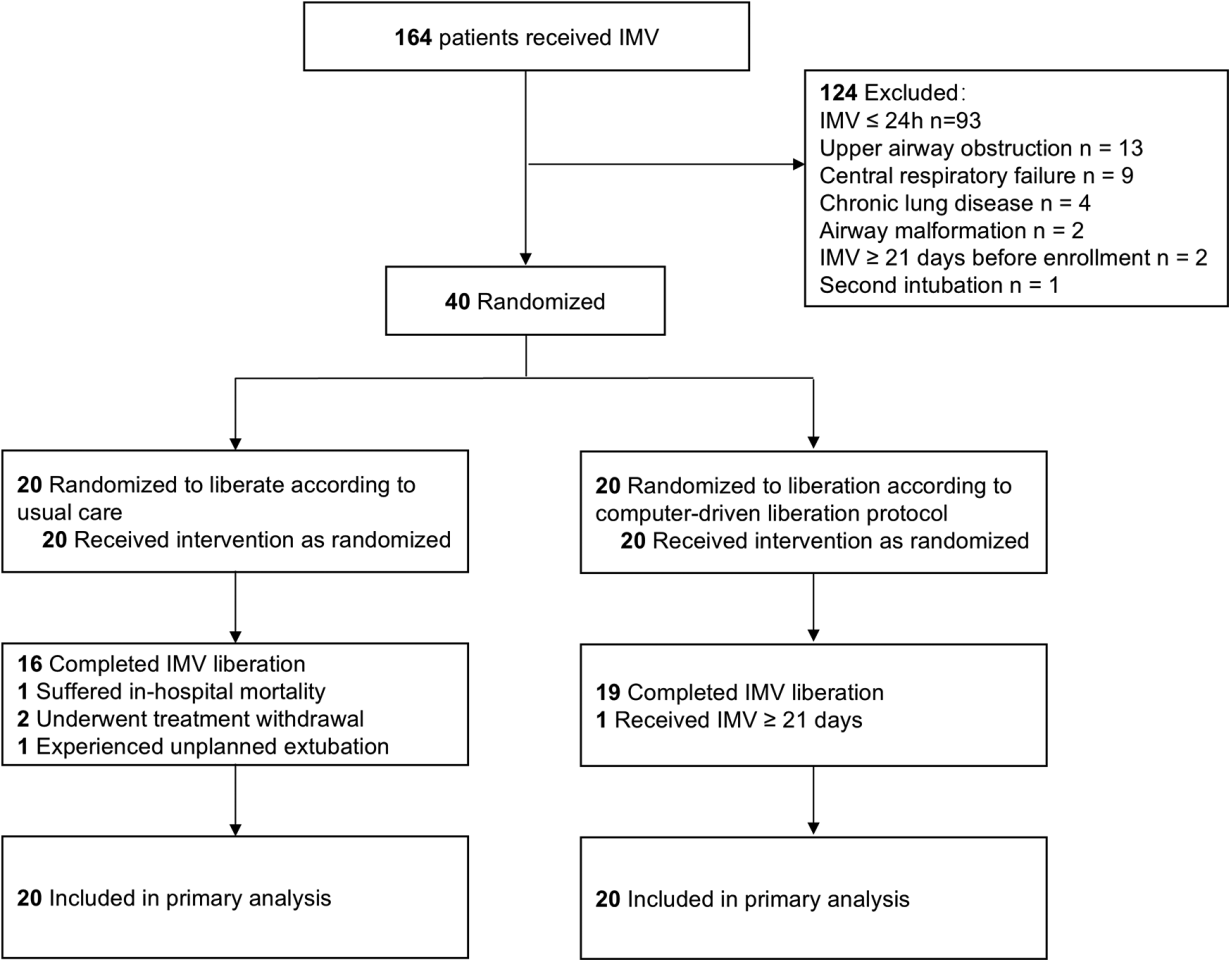
Patient flow. IMV, invasive mechanical ventilation.

A total of 164 patients underwent IMV from April 17, 2023 to June 16, 2023. Seventy-one patients received IMV for more than 24 h. Thirty-one patients were excluded. A total of 40 patients (20 patients for each group) were recruitment, enrolled and randomized (Figure 2) and followed to hospital discharge. Baseline patient characteristics of the study groups were similar (Table 1). Pneumonia and cardiac surgery were the most common indications for IMV. Most patients did not have comorbidities.

**Table 1:** Baseline patient characteristics.

Characteristics	Test group (*n* = 20)	Control group (*n* = 20)	Total (*n* = 40)	*p* value
Age, mo	8.5 (3.0, 51.3)	9.5 (1.25, 30.8)	8.5 (2.3, 33.3)	0.75
Female, *n* (%)	10 (50.0)	14 (70.0)	24 (60.0)	0.20
Severity of illness				
PaO_2_/FiO_2_	171.7 (139.3, 261.0)	213.9 (155.2, 311.1)	193.3 (148.8, 297.1)	0.75
OI	4.9 (3.0, 8.3)	5.2 (2.9, 8.3)	4.9 (2.9, 8.3)	0.75
PRISM III	12.5 (10.0, 15.0)	12.5 (10.0, 15.8)	12.5 (10.0, 15.0)	0.75
PELOD-2	7.0 (3.0, 8.0)	6.0 (5.0, 7.0)	6.5 (3.0, 7.8)	0.75
Indication for ventilation, *n* (%)				0.84
Pneumonia	10 (50.0)	9 (45.0)	19 (47.5)	
Cardiac surgery	7 (35.0)	6 (30.0)	13 (32.5)	
Others	3 (15.0)	5 (25.0)	8 (20.0)	
Comorbidities, *n* (%)				0.95
None	12 (60.0)	10 (50.0)	22 (55.0)	
Congenital heart disease ^a^	4 (20.0)	6 (30.0)	10 (25.0)	
Genetic disorders	2 (10.0)	2 (10.0)	4 (10.0)	
Other	2 (10.0)	2 (10.0)	4 (10.0)	

OI, oxygenation index; OI = [MAP × FiO_2_/PaO_2_] × 100.

^a^ Only those patients before surgery for congenital heart disease.

### 3.2 Outcomes

Four control patients did not undergo standard IMV liberation. One patient suffered in-hospital mortality, 2 underwent treatment withdrawal, and 1 experienced unplanned extubation. One test group patient received IMV for more than 21 days and did not undergo extubation during the follow-up period. A total of 35 patients completed IMV liberation [16 (80%) control patients, 19 (95%) test patients]. No patient liberated by the attending physician before the advice by ICCA in the test group. Two patients in the test group were not liberated from IMV after the first ICAA recommendation because the attending physician assessed that their clinical status precluded liberation. One patient in the test group experienced extubation failure caused by postextubation upper airway obstruction within 24 h.

Three investigators were fully proficient in all aspects of the research protocol. All chief nurses were competent in the correct use of ICCA and could coordinate investigators to complete the process during routine clinical practice. All predetermined parameters were fully recorded.

**Figure 3:**
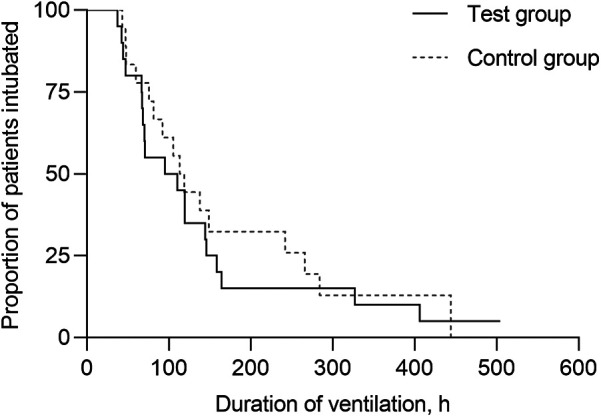
Kaplan–Meier curves of the probability remaining on invasive mechanical ventilation. The median duration of IMV was 95.3 h (95%CI, 9.07–181.53) in the test group and 113.3 h (95%CI, 85.90–140.70) in the control group, without significant difference (*p* = 0.62).

Kaplan–Meier curves of the probability of continued IMV are shown in Figure 3. Median durations of IMV were 95.3 h (95%CI, 9.07–181.53) in the test group and 113.3 h (95%CI, 85.90–140.70) in the control group, with inconclusive effect [hazard ratio [HR]= 1.18 [95%CI, 0.61–2.31]; *p* = 0.62]. There were inconclusive effect in use of systemic glucocorticoid therapy [75.0% vs. 50.0%; relative risk [RR] = 1.50 [95%CI, 0.90–2.49]; *p* = 0.10]; noninvasive respiratory support [35.0% vs. 25.0%; RR = 1.40 (95%CI, 0.53–3.68); *p* = 0.49]; PICU LOS [6.9 days [95%CI, 5.00–8.86] vs. 7.0 days [95%CI, 5.58–8.40]; HR = 1.12 [95%CI, 0.57–2.21]; *p* = 0.74]; and hospital LOS [22.9 days [95%CI, 11.48–34.24] vs. 26.9 days [95%CI, 17.86–35.94]; HR = 0.69 [95%CI, 0.34–1.41]; *p* = 0.31] between the control and test groups, respectively.

### 3.3 Ventilator parameters

Pre-liberation values of PIP, PEEP, MAP, FiO2, PaO2/FiO2 and OI were similar between the two groups (Table 2).

**Table 2:** Ventilator parameters before liberation.

Parameters	Test group (*n* = 19)	Control group (*n* = 16)	Total (*n* = 35)	*p* value
PIP, cmH_2_O	19.0 (16.0, 21.0)	17.5 (15.3, 20.0)	18.0 (16.0, 21.0)	0.58
PEEP, cmH_2_O	5.0 (5.0, 5.5)	5.0 (5.0, 6.0)	5.0 (5.0, 6.0)	0.73
MAP, cmH_2_O	9.3 (8.5, 10.0)	9.5 (8.4, 10.0)	9.4 (8.5, 10.0)	0.66
FiO_2_, %	40.0 (35.0, 45.0)	35.0 (30.5, 40.0)	40.0 (35.0, 40.0)	0.90
PaO_2_/FiO_2_	228.0 (179.8, 270.0)	265.7 (220.1, 317.7)	233.2 (202.0, 280.0)	0.62
OI	4.2 (3.2, 5.1)	3.8 (2.8, 4.5)	4.1 (3.0, 4.7)	0.39

PIP, peak inspiratory pressure; PEEP, positive end-expiratory pressure; MAP, mean airway pressure; FiO_2_, fraction of inspired oxygen; OI, oxygenation index, OI = [MAP × FiO_2_/PaO_2_] × 100.

## 4 Discussion

We conducted this 2-month pilot study validated the feasibility of applying our computer-driven decision support tool to pediatric patients; the feasibility of conducting a larger prospective trial of our study protocol with adequate statistical power to assess the efficacy of the intervention; and demonstrated successful training of PICU personnel. Our team of investigators mastered the application of the protocol. However, our statistical analysis predicted that an enrollment period of 20 months would be needed to enroll the calculated sample size.

We adopted screening criteria by combining those of previous studies (12, 28–30) and those learned from our clinical experiences. The first issue was whether ventilator criteria were too high. Previous study protocols adopted different criteria. Higher screening criteria may necessitate earlier patient assessments, which may increase staff workload, but might identify candidates for earlier liberation and thereby shorten the duration of IMV. Because of the potential benefits of reduced IMV duration, we adopted current criteria. The combining of our protocol with current clinical practice algorithms did not add an excessive work burden. Except for the patient who received IMV for more than 21 days, all patients in the test group completed our protocol and were liberated, and 84.21% (16/19) of patients passed the first SBT. In Fronda et al.'s study (12), 84.33% (113/134) of patients passed the first SBT, and only one patient required more than two SBTs. Most patients who met the screening criteria subsequently passed the SBT. Compared to clinician driven ventilator liberation protocols, computer-driven protocols reduce the need for repetitive screening. Therefore, implementing rational screening criteria alongside protocols tailored to daily clinical practice can reduce screening fatigue and increase compliance rates.

The next issue was whether the resolution of the indication for intubation should be considered before screening or after completion of the protocol. It was one of the criteria for weaning readiness in the study reported by Mehta et al. (30), but was not incorporated into our protocolized screening because this criterion was assessed subjectively, which may introduce bias. Participation of the attending physician in the test group after completion of the protocol might reduce bias. However, another possibility is that the attending physician might liberate patients before screening in the test group. This is an unavoidable challenge due to the inherent complexity of clinical practice. Both intention-to-treat and per-protocol analyses will be performed in the subsequent larger study.

Owing to the finely designed workflow, repeated program debugging and study simulation conducted prior to the pilot study, all 40 enrolled patients completed follow-up, all predetermined parameters were recorded, and relevant personnel were trained. These results demonstrate that a larger study is feasible in out unit. However, due to the small sample size of this pilot study, unforeseen issues may arise in subsequent research.

There was inconclusive effect of IMV duration, PICU LOS and hospital LOS between patients undergoing either a computer-driven liberation protocol or standard care. Given the limited sample size and inherent variability among the measured parameters, we opted not to perform multivariate adjustments in this preliminary analysis. In our subsequent larger study, we plan to conduct comprehensive multivariate analysis to better elucidate the factors influencing IMV duration. We compared pre-liberation ventilator parameters between the two groups to explore whether the computer-driven protocol could drive patients to liberate from higher ventilator parameters. Patients liberating from higher ventilator parameters may hypothetically undergo earlier liberation. However, we observed no significant difference in pre-liberation ventilator parameters between test and control groups.

Few studies of computer-driven ventilator liberation protocols have been completed among pediatric patients. Jouvet et al. (28) conducted a pilot study (*n* = 30) to assess whether a computer-driven protocol can decrease the duration of IMV compared to usual care, and found no significant differences in duration of IMV, PICU LOS, and hospital LOS between test and usual care groups, respectively.

Studies of protocols driven by physicians, nurses, or respiratory therapists have yielded varying results. The intergroup similarity of outcomes observed in our study might be explained by several factors. First, this was a pilot study with a small sample size that may have limited adjustments for suspected confounding factors. Second, indications for IMV were heterogenous. A multicenter study completed by Blackwood et al. (13) that featured high patient heterogeneity demonstrated that a ventilator liberation protocol significantly shortened the duration of IMV, but with small effect size. The PICU LOS was similar between groups. The hospital LOS was significantly longer in patients undergoing the ventilator liberation protocol. Meanwhile, Foronda et al. (12) demonstrated that a ventilator liberation protocol abbreviated the duration of IMV (from 4.7 days to 3.5 days) in a heterogenous cohort. Mehta et al.'s study (30) reported that a ventilator-weaning pathway decreased the duration of ventilation by a median of 3.6 days without different reintubation rates among pediatric patients with acute respiratory distress syndrome, although PICU LOS was not significantly reduced. Ferreiara et al. (25) compared SBT with physician-led weaning for predicting extubation success in pediatric patients following cardiac surgery. Extubation success was significantly higher (83% vs. 68%) and PICU LOS was significantly shorter (median 85 h vs. 367 h) in patients undergoing SBT compared to controls, respectively. However, durations of IMV and hospital LOS were similar between the test and control groups, respectively.

In summary, studies of patients receiving IMV for single or heterogenous indications have yielded discrepant results. Potential confounding factors should be minimized to the greatest possible extent. In addition, the protocolized screening interval may represent another confounding factor. Computer screened patients at 8:00 AM daily in our study. The time and interval we chosen were most fit for our routine. Most other studies also screened patients daily. Screening at shorter intervals may identify candidates for earlier liberation, but may burden investigators and clinicians. Although patients were screened every 3 h in Loberger et al.'s study (29), durations of IMV and PICU LOS did not differ between pre-intervention and intervention cohorts. In a study of adult patients that compared daily screening to at least twice daily screening led by respiratory therapists, more frequent screening was not associated with shorter duration of IMV or more successful extubation (31). Thus, the effect of screening intervals in ventilator liberation protocols needs further study. The most important limitations of the aforementioned studies were their unblinded study designs, which may have introduced bias but were inevitable given the role of the attending physician in final decision making.

## 5 Conclusions

This pilot study confirmed that the conduction of a large randomized controlled trial of a computer-driven IMV liberation protocol is feasible in our PICU. However, there was inconclusive effect in the durations of IMV between intervention and control groups. A larger prospective trial with adequate statistical power is needed to further explore the utility of a computer-driven IMV liberation protocol.

## Data Availability

The raw data supporting the conclusions of this article will be made available by the authors, without undue reservation.
